# How Should Complicated Cases of Thrombotic Thrombocytopenic Purpura With Positive Coombs Test Be Treated?

**DOI:** 10.7759/cureus.50742

**Published:** 2023-12-18

**Authors:** Moutaz Ghrewati, Anas Mahmoud, Tala Beilani, Karam Zakharia, Mehandar Kumar

**Affiliations:** 1 Hematology and Oncology, St. Joseph’s University Medical Center, Paterson, USA; 2 Internal Medicine, St. Joseph’s University Medical Center, Paterson, USA; 3 Internal Medicine, Kansas City University, Kansas City, USA

**Keywords:** atypical ttp, caplacizumab, coombs positive hemolytic anemia, immune hemolytic anemia, non-immune hemolytic anemia, refractory ttp, rituximab (rtx), therapeutic plasma exchange (tpe)

## Abstract

Thrombocytopenia with concomitant anemia is a serious condition with a high mortality risk. Destruction of platelets, i.e., thrombocytopenia, can be secondary to either auto-antibodies (immune-mediated) or mechanical destruction (non-immune-mediated). The Coombs test is a widespread tool to differentiate between the two categories, resulting in different specific treatment approaches for each diagnosis. A peripheral blood smear can also help make the diagnosis; for instance, in cases of mechanical destruction such as thrombotic thrombocytopenic purpura (TTP), the red blood cell (RBC) shape looks fragmented, forming schistocytes. In rare instances, TTP can present with both schistocytes and a positive Coombs test, challenging the diagnosis of TTP. TTP is a hematological emergency requiring appropriate anticipation and the initiation of treatment prior to the confirmatory ADAMTS-13 test results. Mild forms of TTP can be managed with glucocorticoids and therapeutic plasma exchange. Refractory cases need more aggressive additional treatment with caplacizumab and rituximab. Caplacizumab is an expensive medication that is usually reserved for use after confirmation of a TTP diagnosis. The advantage of caplacizumab lies in its targeted mechanism of action against the A1 domain of the von Willebrand multimers that are normally destructed by the ADAMTS-13 enzyme. Here, we present a young female patient with confirmed TTP, and the initial diagnosis was challenged by the presence of antibodies with the Coombs test. Very little research has studied this rare instance and the appropriate treatment. Our case will save many future lives, as clinicians should be more aggressive in treating refractory TTP with a positive Coombs test.

## Introduction

Thrombotic thrombocytopenic purpura (TTP) is a life-threatening condition that necessitates vigilant anticipation, workup, and empiric treatment before diagnosis confirmation. The pentad of TTP entails microangiopathic hemolytic anemia (MAHA), thrombocytopenia, fever, neurological features, and renal dysfunction [1]. However, the pentad presentation only occurs in some patients, complicating the diagnosis process. Moreover, excluding other causes of thrombocytopenia and hemolytic anemia is mandatory prior to treatment, as different causes require different treatment strategies [1,2]. The highlighting diagnostic feature of TTP is the presence of schistocytes in the peripheral blood smear since the hemolysis is mechanical and not immune-mediated, for which a negative Coombs test is required to diagnose TTP [1,2]. In rare instances of TTP, the Coombs test can be positive due to unknown causes. In such cases, treatment is usually delayed until the ADAMTS-13 test result confirms the diagnosis; however, this test takes more than five days [3]. Mild cases of TTP can allow for first-line therapies such as glucocorticoids and plasma exchange therapy to stabilize the patient until the ADAMTS-13 test comes back, and further treatment can follow [3,4]. Unfortunately, this is not feasible in more severe forms of TTP and end-organ damage can be irreversible, as it is refractory to first-line therapies, and more aggressive treatment is required. Since the platelet destruction in TTP is mechanical, caplacizumab can block the platelet and ultra-large vWF interaction, which prevents the destruction of the platelets [5]. Our patient, presented in this article, had a sporadic form of refractory TTP with a positive Coombs test and unfortunately passed away despite early treatment initiation. Our cases highlight this very scarce form of TTP, which will help future research studies of these cases and the appropriate aggressive treatment needed in these cases.

## Case presentation

A 23-year-old African American female with no medical history presented to the ED for intermittent severe headaches for four days. Vitals were stable, with a blood pressure of 120/70 mmHg, a heart rate of 70 beats per minute, and a respiratory rate of 13, and the patient was not febrile. A complete neurological examination did not show any deficits. A CT scan of the head and CT angiography of the head did not reveal any abnormalities. The blood work results are in Table 1.

**Table 1:** Blood lab work results With pertinent results of platelets counts of 18, Hb level of 6.4, and WBC of 17. BUN: blood urea nitrogen, eGFR: estimated glomerular filtration rate, ALK: anaplastic lymphoma kinase, AST: aspartate transferase, ALT: alanine transaminase, HbA1C: hemoglobin A1C, CRP: C-reactive protein, ESR: erythrocyte sedimentation rate, WBC: white blood cell, RBC: red blood cell, Hgb: hemoglobin, Hct: hematocrit, N/A: not available

Labs	Value	Reference range
Sodium	137 mEq/L	135-145 mEq/L
Potassium	3.5 mEq/L	3.5-5 mEq/L
Chloride	102 mEq/L	98-107 mEq/L
Bicarb	27 mEq/L	21-31 mEq/L
Glucose	115 mg/dl	70-110 mg/dl
Ketones	N/A	Negative
BUN	14 mg/dl	7-23 mg/dl
Creatinine	0.95 mg/dl	0.6-1.3 mg/dl
eGFR	>60 ml/min/1.73 m2	>60 ml/min/1.73 m2
Total bilirubin	1.9 mg/dl	0.3-1.1 mg/dl
Total protein	6.9 g/dl	6.4-8.4 g/dl
Albumin	4.2 g/dl	3.5-5.7 g/dl
ALK phos	57 unit/L	34-104 unit/L
AST	32 unit/L	13-39 unit/L
ALT	32 unit/L	7-52 unit/L
HbA1C	N/A	4-6
Magnesium	1.8 mg/dl	1.7-2.5 mg/dlcrp
CRP	7 mg/L	<9.9 mg/L
ESR	8 mm/hr	<10 mm/hr
WBC	17 28.9 x 10^3/ mm3	4.5-11 28.9 x 10^3/ mm3
RBC	2.01 1.90 x 10^3/mm3	4.3-5.8 1.90 x 10^6/mm3
Hgb	6.4 g/dl	13.5-17.5 g/dl
Hct	19.1%	41-53%
Platelets	18 k/mm3	140-440 k/mm3
Bands	20%	0-10%

**Figure 1:**
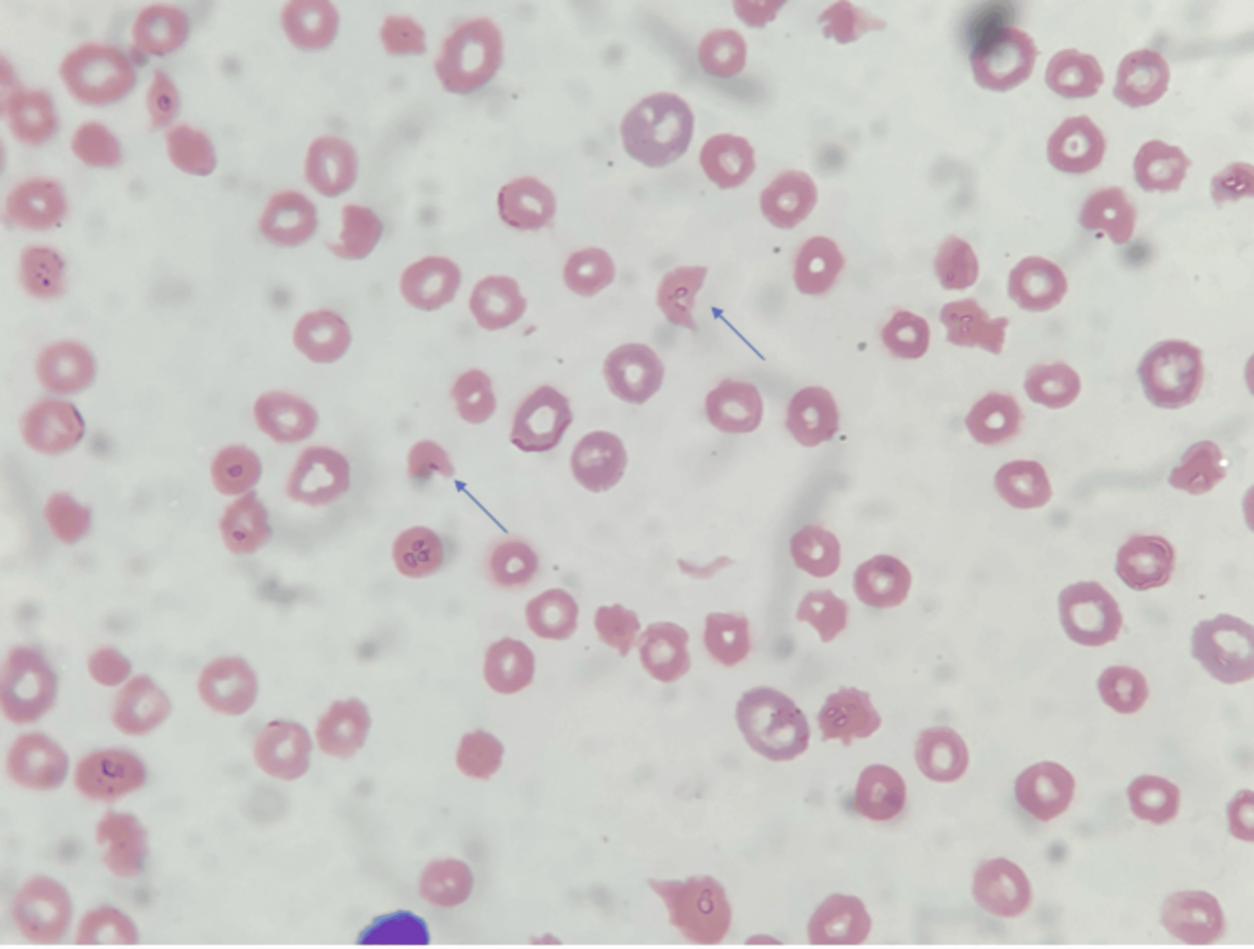
Peripheral blood smear showing schistocytes on peripheral blood smear (blue arrows), which occurs as a result of mechanical destruction occurring in TTP

Upon detailed history evaluation, the patient denied any constitutional symptoms, rash, joint pain, use of fava beans, or any significant recent change in her daily life. The hematologist was consulted, reviewed the blood smear, and identified schistocytes (Figure 1).

Further coagulation study results became available as mentioned in Table 2.

**Table 2:** Results of coagulation studies indicating a hemolytic cause for anemia PT: prothrombin time, INR: international normalized ratio, PTT: partial thromboplastin time, LDH: lactate dehydrogenase

Labs	Value	Reference range
PT	15.6 seconds	12.2-14.9 seconds
INR	1.3	0.5-4 (NA)
PTT	28.6 seconds	21.3-35.1 seconds
Fibrinogen level	289 mg/dl	200-400 mg/dl
D-dimer	2.47 mg/L	<0.5 mg/L
Fibrin degradation product	9 μg/ml	<10 μg/ml
Von Willebrand factor Ag	163 IU/dL	50-200 IU/dL
LDH	1141 U/L	140-270 U/L

With schistocytes on the peripheral smear, TTP was on top of the differential diagnosis. However, unexpectedly, the lab called the medical team, relaying that the Coombs test (IgG) was positive. The patient was transfused with one unit of cross-matched blood, while the workup was focused on investigating possible Evans syndrome. Therefore, the hematologist decided to proceed with methylprednisolone along with blood transfusion (with the avoidance of platelet transfusion as TTP was still a high possibility). The next day, the hematologist reviewed the peripheral smear again, and it still showed the persistence of schistocytes, the hemoglobin level downtrended from 6 gm to 5.5 gm, and the platelet count dropped from 18 to 11. Hence, the probability of TTP further increased, and the ADAMTS-13 level was sent with reflex to antibodies. The critical care team was consulted and accepted a transfer to the medical intensive care unit (MICU). Therapeutic plasma exchange (TPE) was immediately started. Five days later, the ADAMTS-13 result was 2%, and TTP was confirmed. The platelet count modestly improved to 22, and the hemoglobin level only increased to 7.2 gm with daily sessions of TPE; hence, the hematologist decided to increase the TPE sessions to twice daily. Meanwhile, caplacizumab was ordered and arranged by the MICU pharmacy.

Unfortunately, on the sixth day prior to caplacizumab administration, the patient’s clinical status was deteriorating, making refractory TTP the most likely diagnosis. The patient developed a generalized tonic seizure and was emergently intubated for airway protection. A few hours later, the patient became hypotensive and hypoxic. Bedside point-of-care ultrasound (POCUS) showed an overall decrease in global contractility, with a positive McConnell sign suggesting a massive pulmonary embolism. A tissue plasminogen activator was administered; meanwhile, norepinephrine, vasopressin, and phenylephrine were initiated consequently to maintain adequate blood pressure. Minutes later, the patient lost her pulse and suffered a cardiac arrest. The return of spontaneous circulation was achieved. The patient, unfortunately, suffered two consequent cardiac arrests, for which cardiothoracic surgery was consulted for possible mechanical thrombectomy vs. extracorporeal membrane oxygenation. However, the patient was deemed unstable for any intervention, and the patient passed away.

## Discussion

Hemolytic anemia refers to any drop in the hemoglobin levels secondary to the hemolysis of red blood cells. Hemolytic anemia can be classified into two categories based on the defective factors that lead to hemolysis. Intrinsic factors include hemoglobinopathies (e.g., sickle cell disease and thalassemias), membrane defects (e.g., spherocytosis), and enzyme defects (e.g., G6PD deficiency) [1], whereas extrinsic factors include immune hemolytic anemia (IHA) (warm vs. cold). Other causes include, but are not limited to, medications, infections (e.g., malaria and babesiosis), or thrombotic microangiopathy (TTP, disseminated intravascular coagulation, atypical hemolytic uremic syndrome, mechanical valves, and medications). Furthermore, hemolytic anemia can be classified based on the location of hemolysis as intravascular or extravascular. In extravascular hemolysis, the destruction occurs outside the blood vessels, e.g., the spleen or the liver, and the peripheral smear should typically show spherocytes and also erythrocyte agglutination if the blood is not warmed upon collection [1]. On the other hand, intravascular hemolysis could be secondary to cell membrane damage, yielding characteristic RBC fragments (schistocytes) on the peripheral smear. Intravascular hemolysis is more likely to occur with low serum levels of haptoglobin and urinary hemosiderin. Both extravascular and intravascular would demonstrate raised unconjugated bilirubin, reticulocyte count, and normocytic or macrocytic anemia. However, all these blood work results should be interpreted as a group and in context for the patients, as any individualized interpretation could be misleading, which is evident in our case as our patient tested positive for a direct antiglobulin test (DAT), indicating autoimmune hemolytic anemia (AIHA), and at the same time, the peripheral smear showed schistocytes, indicating TTP. Moreover, lactate dehydrogenase is not a specific enzyme and can occur secondary to many diseases, including hemolysis, cancer, and other conditions. Liver diseases could also cause low haptoglobin levels. Reticulocytopenia can occur in cases of severe nutrient deficiency, such as vitamin B12 deficiency, which leads to ineffective hematopoiesis. AIHA should be appropriately managed as the host’s immune system attacks against its own red cell antigens, resulting in decompensated acquired hemolysis. AIHA can be subclassified into warm and cold subtypes, and the hallmark is positivity to the Coombs test [1]. The diagnosis of warm AIHA is established with positive Coombs against IgG with or without positive complement C3. On the other hand, DAT will be positive only against complement C3 or IgM, but not against IgG, in cold agglutinin disease (i.e., cold immune hemolytic anemia) [1].

On the other hand, TTP is defined by low platelet numbers secondary to a shearing process rather than auto-antibodies against the platelets. Diagnostic features of TTP include thrombocytopenia, MAHA, neurogenic abnormalities, renal abnormalities, and fever. At the same time, diagnosis requires the absence of alternative etiologies for both thrombocytopenia and MAHA [2]. Two significant principles are needed for TTP to occur: firstly, antibodies against ADAMTS-13 that decrease its activity, and secondly, a trigger that induces systemic endothelial cell damage [3]. Endothelial damage with resultant von Willebrand factor (vWF) multimers is supra-adhesive to platelets, and the decreased activity of ADAMTS-13 results in systemic platelet agglutination and thrombosis. [4]. Hence, the diagnosis of TTP is confirmed by low levels of vWF-cleaving protease (ADAMTS-13). This test is very complex, and the results take a long time, necessitating early anticipation and treatment initiation. However, a study showed that the level of ADAMTS-13 may not help diagnose TTP in many cases [6]. Mannucci et al. [7] showed that low levels of ADAMTS-13 are found in a significant portion of cases of systemic lupus erythematosus (SLE) and other systemic connective tissue diseases, highlighting the risk of TTP in SLE patients and the importance of the rare TTP cases with a positive Coombs test. Our patient tested positive for anti-nuclear antibodies, but unfortunately, further SLE testing was not applicable due to the rapid deterioration of the patient’s clinical status.

Most cases of TTP are idiopathic without an identifiable cause or associated illness. TTP can also happen during pregnancy. Infections and drugs, e.g., ticlopidine and cyclosporine A, can also cause TTP. In TTP, hemolysis is a secondary shearing force rather than auto-antibodies; therefore, the DAT test is negative in almost every TTP case and should remain negative to establish a TTP diagnosis. A positive DAT test is usually linked to immune hemolytic anemia. Rare causes such as blood transfusion or intravenous immunoglobulin administration can cause positive coombs or DAT in the absence of immune hemolytic anemia, and neither of them occurred in our patient. However, very few cases have reported positive DAT tests in TTP cases. A child tested positive for coombs and the platelets did not respond to glucocorticoids initially; however, after considering TTP, the platelets responded dramatically to plasma exchange. [8]. Aleem et al. [9] conducted the most extensive series from a single center on six patients with TTP and positive Coombs. They concluded that a positive Coombs test is not against the diagnosis of TTP. They also highlighted the association between TTP and SLE. While a positive direct Coombs test is against the diagnosis of TTP, three SLE patients had a weakly positive direct Coombs test along with apparent features of TTP. They also found that four patients responded to plasmapheresis only, and only one required cytotoxic therapy. Due to the rarity of this coincidence, it is challenging to propose an incidence or prevalence.

The fatality rate is high in TTP; therefore, early diagnosis and treatment are crucial. Plasma exchange has dramatically changed TTP prognosis and made it a curable illness, and it is considered the first-line treatment for TTP. The presence of primary diagnostic criteria (thrombocytopenia, MAHA with negative DAT, presence of schistocytes, and presence of abnormal renal functions (proteinuria, hematuria, acute renal failure), change in mental status, fever [high fever with chills is against TTP], and abdominal symptoms (nausea, vomiting, diarrhea) are sufficient to start TPE. Sayani et al. extensively discussed the possible treatment options for refractory TTP [10]. Refractory TTP is defined as a failure of the platelet count to go up after four to seven days of TPE or any clinical deterioration in a patient already receiving standard therapy. It is imperative to reevaluate the condition for other causes of thrombocytopenia and AIHA that may need additional therapy. For instance, the initial response to TPE with subsequent deterioration may be due to underlying sepsis or drug-induced thrombocytopenia, such as newly started antibiotics. There is minimal literature regarding the management of refractory TTP. As corticosteroids are started at a dose of 1 mg/kg per day in all TTP patients, a randomized trial illustrated the importance of increasing the dose to 10 mg/kg per day for three days in refractory cases of TTP, especially with neurological symptoms [11]. Increasing the frequency of TPE from once daily to twice daily has also been studied; however, the results failed to show clinical significance [12]. Further research on the efficacy of TPE in TTP patients with positive Coombs can improve the outcome of such rare cases. Rituximab is a chimeric anti-DC20 monoclonal antibody, is very efficient in treating refractory TTP, and has decreased both replacements and platelet recovery time in refractory TTP [13]. However, it takes time to demonstrate the effect, and more literature has yet to discuss its use in acutely severely deteriorating patients. The recommended dose is 375 mg/m2 once a week for four weeks. Splenectomy has shown effectiveness in treating refractory TTP, with only 8% of patients failing to respond to splenectomy [14]. However, surgeons will perform splenectomy in clinically stable patients, which was not the case in our sick patients, unfortunately. Cyclosporine, an immunomodulating drug that inhibits T-cell activation and interleukin-2 production, has successfully treated refractory TTP; however, its use is rare now because it can also cause thrombotic microangiopathy, independent of TTP [10]. The use of both cyclophosphamide and vincristine has been effective in aborting refractory TTP, but their extensive side effects limit their use nowadays. Bortezomib, a proteasome inhibitor mainly used in multiple myeloma, has been reported to be used in refractory TTP; however, further studies are required to guide physicians to its use [15]. N-acetylcysteine has been recently tested in treating refractory TTP as it could substitute ADAMTS-13 and reduce the size of ultra-large vWF multimers; however, the use is still under clinical trials and needs further studies to support its effectiveness in treating refractory TTP. Recombinant ADMATS-13 might be the future therapy for refractory TTP; however, it also needs more studies. Caplacizumab is a monoclonal antibody fragment that can block the interaction between vWF and platelets within four to six hours, which is the interaction that promotes thrombosis in TTP. Rapid blocking of ongoing thrombosis is the most critical step in refractory TTP; fortunately, caplacizumab can achieve it. Caplacizumab is the only FDA-approved medication for managing acquired TTP. It can be used as routine management in all cases of TTP; however, it is the most expensive medication in treating TTP, with a cost ranging from 300 thousand to 1.5 million US dollars; therefore, it is usually preserved for critically ill patients who are not responding to TPE and have neurological symptoms [5]. It is essential to understand that caplacizumab only prevents the interaction between platelets and the ultra-large vWF multimers and does not treat the underlying cause of TTP; hence, the concurrent use of TPE and rituximab to remove or decrease the production of antibodies, respectively. Soon, when caplacizumab's cost is reasonable, refractory TTP mortality will decrease significantly [16].

## Conclusions

Thrombocytopenia can be isolated or occur with a simultaneous decrease in hemoglobin level, as in hemolytic anemia. On a peripheral smear, extravascular hemolysis has a spherocyte shape, while intravascular hemolysis has schistocytes, as in TTP. Antibodies against ADAMTS-13 lead to vWF multimers, which activate the endothelium, resulting in hemolysis and platelet destruction. The ADAMTS-13 level test is very time-consuming and can result in five days, which puts significant danger on TTP patients if the appropriate treatment is delayed. Hence, TTP should be urgently treated on suspicion. The Coombs test can differentiate between immune and non-immune hemolytic anemia. A TTP diagnosis is more likely when the Coombs test is negative. Positive Coombs tests in TTP patients are very rare, which makes the diagnosis and treatment very challenging. Our patient presented with typical features of TTP, but due to a positive Coombs test, aggressive treatment was delayed. TTP should be considered even with a positive Coombs test, and aggressive treatment, e.g., a high dose of glucocorticoid, more frequent therapeutic plasma exchange, rituximab, and caplacizumab, should not be delayed when the count of platelets is not responding to glucocorticoid therapy.
